# Assessment of Key Factors Impacting Variability in AAV Vector Genome Titration by Digital PCR

**DOI:** 10.3390/ijms25105149

**Published:** 2024-05-09

**Authors:** Guangyu Wang, Qiang Ma, Changlong Wei, Lei Yu, Hua Bi, Jing Jin, Xi Qin, Yong Zhou, Junzhi Wang

**Affiliations:** NHC Key Laboratory of Research on Quality and Standardization of Biotech Products, National Institutes for Food and Drug Control, Beijing 100050, China; wanggy@nifdc.org.cn (G.W.); maqiang@nifdc.org.cn (Q.M.);

**Keywords:** AAV, rAAV, genome titer, dPCR, variation, pretreatment

## Abstract

Recombinant adeno-associated virus (rAAV) has emerged as a prominent vector for in vivo gene therapy, owing to its distinct advantages. Accurate determination of the rAAV genome titer is crucial for ensuring the safe and effective administration of clinical doses. The evolution of the rAAV genome titer assay from quantitative PCR (qPCR) to digital PCR (dPCR) has enhanced accuracy and precision, yet practical challenges persist. This study systematically investigated the impact of various operational factors on genome titration in a single-factor manner, aiming to address potential sources of variability in the quantitative determination process. Our findings revealed that a pretreatment procedure without genome extraction exhibits superior precision compared with titration with genome extraction. Additionally, notable variations in titration results across different brands of dPCR instruments were documented, with relative standard deviation (RSD) reaching 23.47% for AAV5 and 11.57% for AAV8. Notably, optimal operations about DNase I digestion were identified; we thought treatment time exceeding 30 min was necessary, and there was no need for thermal inactivation after digestion. And we highlighted that thermal capsid disruption before serial dilution substantially affected AAV genome titers, causing a greater than ten-fold decrease. Conversely, this study found that additive components of dilution buffer are not significant contributors to titration variations. Furthermore, we found that repeated freeze–thaw cycles significantly compromised AAV genome titers. In conclusion, a comprehensive dPCR titration protocol, incorporating insights from these impact factors, was proposed and successfully tested across multiple serotypes of AAV. The results demonstrate acceptable variations, with the RSD consistently below 5.00% for all tested AAV samples. This study provides valuable insights to reduce variability and improve the reproducibility of AAV genome titration using dPCR.

## 1. Introduction

Adeno-associated virus (AAV), a small nonenveloped virus composed of an icosahedral protein capsid and an inner single-stranded genome DNA, has emerged as a promising gene delivery vector in the gene therapy field. Recombinant AAV (rAAV) holds several advantages, such as broad tropism, minimal immunogenicity, and the ability to achieve long-term transgene expression [1,2], which have translated into tangible success in commercial gene therapy applications. Considering the presence of nontherapeutic empty capsids in rAAV products, the genome titer’s preferred use is to characterize rAAV preparations rather than capsid titers. The accurate and reproducible determination of AAV vector genome titer is critical for pharmaceutical analysis, as well as preclinical and clinical dosing. The advent of digital PCR (dPCR) introduced a powerful technique in AAV genome titration, as it is able to absolutely quantify DNA copies without the need for standard curves, and it performs better than traditional quantitative real-time PCR (qPCR) in terms of accuracy and precision [3,4,5]; thus, it has become a preferred alternative to qPCR and even the tool of choice for genome titration.

**Figure 1:**
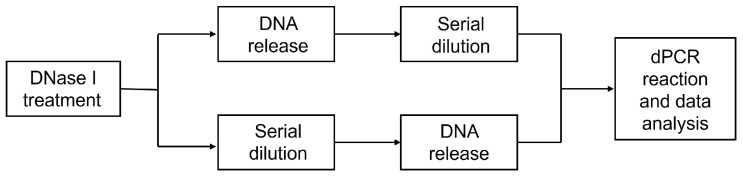
General procedures of the AAV genome titration protocol.

As shown in Figure 1, the AAV samples have to be treated with DNase I first before genome titration for the purpose of digesting unencapsidated DNA and preventing aggregation of viral particles. The following step is the serial dilution of digestion reaction systems, aiming to satisfy the recommended dPCR concentration range. Surfactants such as Pluronic F-68 and blocking agents like sheared salmon sperm DNA are commonly added to the dilution buffer to facilitate the dissolution and dispersion of viral particles. Capsid lysis and genome DNA release are usually performed after serial dilution [4,5,6,7] or could be carried out before dilution. Viral genome extraction before dilution and quantification has also been seen in previous studies [8,9,10]. Finally, dPCR reaction and copy number analysis can be run on various instruments based on different principles. Despite the overall similarities in vector genome titration protocols, subtle differences exist [4,5,6,7,8,9,10], potentially contributing to variations in vector genome titer determination. Such discrepancies pose challenges in comparing and standardizing dosing between studies or laboratories, potentially impacting the safety and efficacy profiles of rAAV in clinical trials.

This study systematically evaluated the impact of various factors in experimental procedure on titration accuracy and robustness, conducting single-factor experiments with AAV5 and AAV8 samples which embed enhanced green fluorescence protein (eGFP) as the gene of interest (GOI). The investigated factors included genome extraction or not, various dPCR instruments, DNase I treatment conditions, additives and their concentrations in the dilution buffer, the sequential order of key steps, etc. The optimized genome titration protocol was subsequently validated with additional samples of multiple serotypes to ensure broad applicability. Exploring factors that may influence genome titration provides insights to mitigate variability and enhance the precision of AAV genome titration using dPCR.

## 2. Results

### 2.1. Comparison between Genome Extraction Protocol and Nonextraction Protocol

Quantifying rAAV vector genome titers can be achieved with or without prior genomic DNA extraction from the viral suspension [6,7,8,9,10]. This raises a fundamental inquiry: Is the extraction of viral genome DNA really necessary? We compared the quantitative results obtained from methods with genome extraction and without extraction; as delineated in Table 1, the precision gauged by the relative standard deviation (RSD) of three independent tests for both AAV5 and AAV8 samples were better in the nonextraction protocol compared with extraction protocol. The variation in AAV8 titers measured by genome extraction protocol was surprisingly up to 28.54%; in addition, AAV5 titers measured by extraction protocol were notably lower than those measured by nonextraction protocol (1.01 × 10^12^ vs. 1.38 × 10^12^ vg/mL), which may be explained by the insufficient recovery of genome DNA by experimental operations and viral DNA extraction reagent kits. Therefore, based on superior precision and recovery, we advocate the nonextraction pretreatment protocol for AAV genome titration.

### 2.2. Checking Titer Variations across Different Instruments

**Figure 2:**
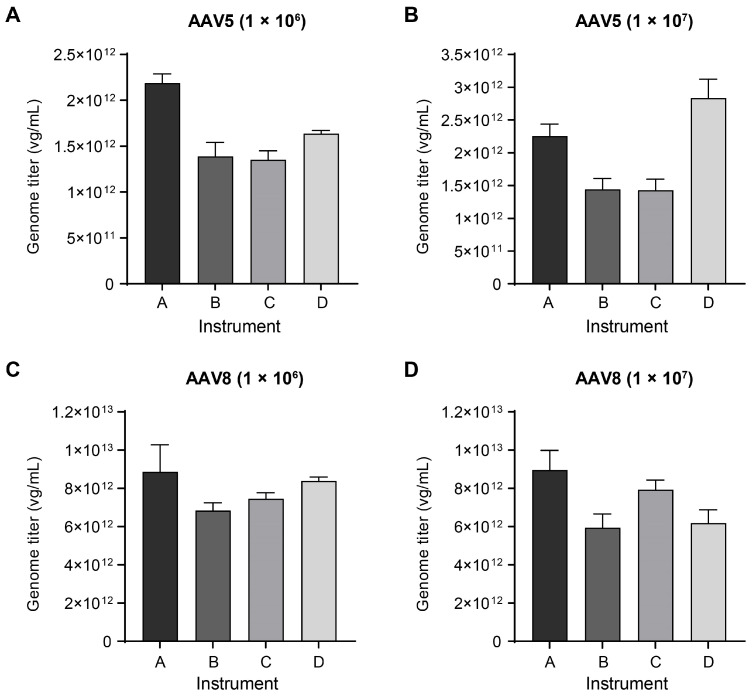
Divergence across dPCR instruments tested with AAV5 and AAV8 samples. The genome titers of AAV5 (**A**,**B**) and AAV8 (**C**,**D**) samples determined by four brands of instruments with the same pretreatment procedure; both samples were diluted and tested at dilution factor of 1 × 10^6^ (**A**,**C**) and 1 × 10^7^ (**B**,**D**). The genome titers were converted values after multiplying the original measured results by the corresponding dilution factors. For each instrument, three experiments were performed for each AAV sample with each dilution, and three technical replicates were set for each test, resulting in a total of nine data points per column. Data are presented as mean with standard deviation (SD). Statistical significance (t-test) was determined between every two instruments for each AAV sample and each dilution. There were significant differences between every comparison except for instruments B and C in (**A**) and (**B**), instruments A and D in (**C**), instruments B and D in (**D**).

Various commercialized dPCR instruments based on different principles have been available for the absolute determination of AAV genome titers. To evaluate the consistency of titer measurement across various instrument platforms, we selected four distinct brands of instruments, including droplet-based (QX200 from Bio-Rad, Naica dPCR system from Stilla) and microwell-based ones (QuantStudio Absolute Q from Applied Biosystems, QIAcuity One from QIAGEN), to quantify the genome of the same AAV5 sample and AAV8 sample with the pretreatment procedure without genome extraction. To avoid any commercial bias, we randomly assigned the letters A, B, C, and D as codes to the four brands of instruments. Different instruments reported divergent genome titers for both AAV samples at the two dilution factors (1 × 10^6^ and 1 × 10^7^) and showed considerable variations (Figure 2). More similar results were observed in the condition of low dilution factor, but the results have a greater difference when the samples were in high dilution factor (RSD within instruments was 23.47% vs. 34.27% for AAV5 and 11.57% vs. 19.89% for AAV8).

In contrast, when the four instruments were employed to determine the genome titers of AAV5 and AAV8 samples using the same batch of genome DNA extractive, the results from different instrument platforms were closely aligned (Figure S1), with RSD within instruments ranging from 4.73% to 8.50%. Therefore, these findings suggested inherent differences among different brands of instrument platforms in quantifying the same AAV suspension sample without DNA extraction. Additionally, caution should be taken when comparing AAV genome titers obtained from different laboratories or using different instruments.

To investigate the effect of pretreatment protocols on the precision and accuracy of AAV genome quantification and avoid instrument-to-instrument variability, we used a single dPCR instrument (QuantStudio Absolute Q, Applied Biosystems) for the remainder of this study.

### 2.3. Testing DNase I Treatment Time and Inactivation

**Figure 3:**
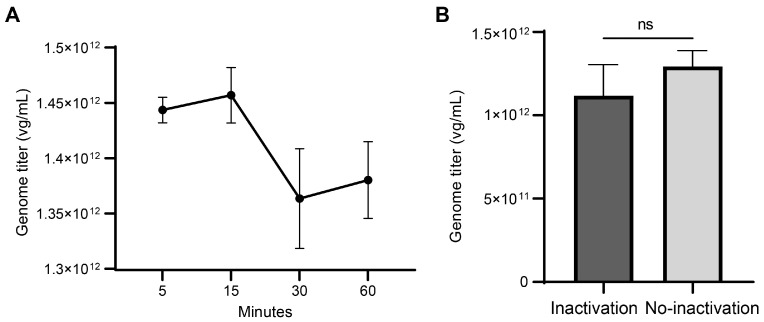
Evaluation of different conditions about DNase I treatment: (**A**) The genome titers of AAV5 sample determined by dPCR after different DNase I incubation time. Data are from three independent replicates for each condition, error bars represent ±1 SD. (**B**) The genome titers of AAV5 sample determined by two pretreatment protocols that were inactivating DNase I by incubating at 80 °C for 10 min after digestion versus no special DNase I inactivation. Data are shown as mean with SD (n = 3). Statistical significance was determined by Mann–Whitney test; “ns” is for not significant.

The first step in AAV genome titration is the digestion of unencapsidated DNA with DNase I. To explore the feasibility of a more time-saving protocol, we set up four parallel groups with different DNase I treatment durations, 5, 15, 30, and 60 min. Results indicate that titers were higher in the 5 and 15 min treatment groups than those from the 30 and 60 min groups. Furthermore, titers stabilized after 30 min incubation (Figure 3A). This trend can be attributed to insufficient digestion of unencapsidated DNA with shorter incubation time. Hence, a DNase I treatment time exceeding 30 min is deemed necessary for accurate measurement of AAV genome titer, consistent with other established protocols [4,5,6,7,9].

In a typical protocol, DNase I remains active until thermal capsid lysis by heating to 95 °C for 10 min before the amplification program in a thermal cycler. To investigate whether residual enzymes can digest released AAV genome during the heating process, potentially causing lower titer, we introduced an additional heating step at 80℃ for 10 min immediately after DNase I treatment to inactivate DNase I adequately only (according to manufacturer’s specification), and this temperature does not approach the melting curve of AAV5 capsid [11]. Surprisingly, compared with no-inactivation, the condition with additional DNase I inactivation presented slightly lower genome titers, although the difference was not statistically significant (Figure 3B). This suggested that deliberate heating inactivation of DNase I postdigestion is unnecessary and not advisable. Furthermore, considering the low concentration of residual DNase I after serial dilution, it poses a negligible threat to the integrity of the AAV genome.

### 2.4. Dilution Buffer Components Do Not Cause Variations in Genome Titers

**Figure 4:**
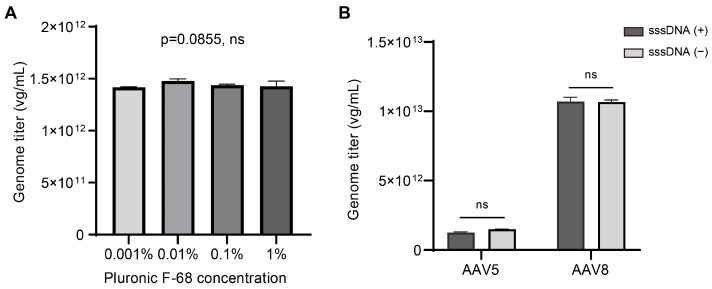
Effects of additive components in dilution buffer on titration: (**A**) The genome titers of AAV5 sample determined by dPCR using dilution buffers with different concentrations of Pluronic F-68. Data are presented as mean with SD. Statistical significance was determined by Kruskal–Wallis test. (**B**) The genome titers of AAV5 and AAV8 samples were determined parallelly on the same digital PCR plates with two buffer formulations that were with the addition of sssDNA or not. Data are presented as mean with SD. Statistical significance was determined by Mann–Whitney test; “ns” is for not significant.

To explore the effects of additive components in dilution buffers, such as Pluronic F-68 (a nonionic surfactant at 0.1% used to enhance dissolution and dispersion of viral particles) and sheared salmon sperm DNA (sssDNA, an effective blocking agent to reduce nonspecific adsorption of AAV particles onto consumable surfaces), we conducted parallel experiments with varying concentrations of these components in the dilution buffer. The AAV5 genome titers did not show significant differences across the four groups with different concentrations of F-68 (Figure 4A), consistent with previous findings [6]. In addition, when comparing results from dilution buffers with and without sssDNA, we found no significant differences in genome titers for both AAV5 and AAV8 samples (Figure 4B). In light of these results, it was suggested that AAV genome titration is not sensitive to the two common additive components in dilution buffer, while it is worth noting that other AAV serotypes may require further investigation.

Based on these findings, we recommend following established protocols [6], with a final concentration of 0.1% for Pluronic F-68 and including sssDNA in the dilution buffer at a final concentration of 2 ng/μL.

### 2.5. Testing the Order of Serial Dilution and Thermal Capsid Lysis

**Figure 5:**
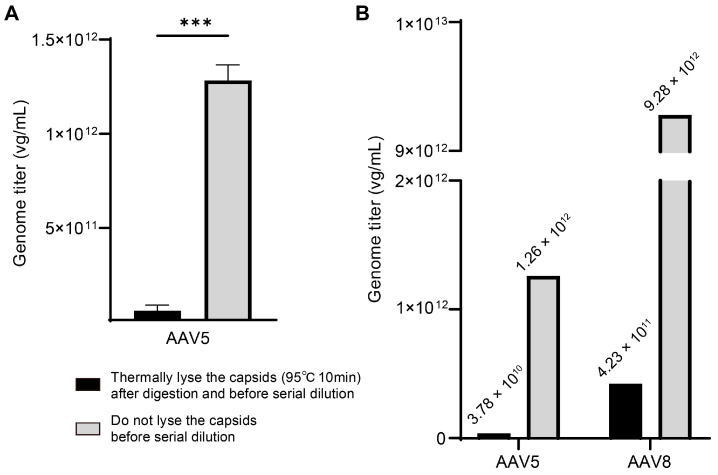
The effect of early capsid lysis on titration accuracy: (**A**) The genome titers of AAV5 sample determined by two pretreatment protocols, which were thermal capsid lysis prior to serial dilution versus thermally lysing capsids after. Data are shown as mean with SD (n = 3). Statistical significance was determined by Mann–Whitney test, ***: *p*-value < 0.001. (**B**) The genome titers of AAV5 and AAV8 samples determined parallelly by the two pretreatment protocols as in (**A**). The average titer values are shown above the columns.

In most protocols of AAV genome titer measurement, DNase I treatment is immediately followed by a serial dilution step, in which intact viral particles are diluted, and then capsids in the PCR reaction system are thermally lysed in the predenaturation step of the PCR program. We next aimed to determine whether altering the order of serial dilution and thermal capsid lysis could lead to variations in titration. We compared a protocol with thermal capsid lysis (95 °C, 10 min) occurring before dilution, in which released genome DNA was serially diluted in theory to the default protocol. Surprisingly, the genome titer of the AAV5 sample from the testing protocol was extremely reduced compared with the default protocol (Figure 5A). We also performed the same experiment with AAV5 and AAV8 samples simultaneously, and both samples exhibited the same phenomenon: the genome titer decreased sharply by more than ten-fold when the capsids were lysed thermally prior to serial dilution (Figure 5B). This finding suggested that changing the sequence of key steps in genome titration protocol is not advisable, as it can lead to substantial variations in quantification results.

### 2.6. Freeze–Thawing Cycles Compromised AAV Genome Titers

**Figure 6:**
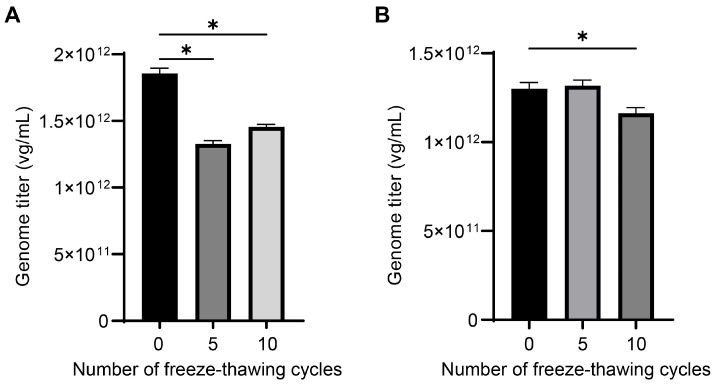
The effect of repeated freeze–thawing on titration. The genome titers determined by dPCR for AAV5 samples in the form of freeze-dried (**A**) and liquid (**B**) that experienced 0, 5, and 10 cycles of freezing and thawing. Data represent mean with one SD of four independent replicates. Statistical significance was determined by Mann–Whitney test, *: *p*-value < 0.05.

Multiple freeze–thawing cycles could lead to lower AAV genome titers and lower qualities in general, particularly during prolonged storage or under varied storage conditions in laboratory settings. To evaluate the impact of repeated freeze–thawing on genome titers, we assessed the genome titers of AAV5 samples which experienced 0, 5, and 10 cycles of freezing and thawing. Our findings revealed a significant decrease in genome titers for both reconstituted freeze-dried AAV and AAV in buffers as the number of freeze–thawing cycles increased, as shown in Figure 6. Based on these results, we recommend limiting the freeze–thaw cycles of viral samples to one for the accurate genome titration of AAV vectors.

### 2.7. Validation of AAV Genome Titer Assay Using dPCR

The optimized digital PCR titration protocol includes the following steps and principles based on our aforementioned results: (1) Use the AAV samples that have not experienced repeated freeze–thawing cycles and adopt the pretreatment procedure without genome extraction. (2) More than 30 min of DNase I treatment is necessary, and there is no need for thermal inactivation after DNase I treatment. (3) Use the dilution buffer with the addition of 0.1% Pluronic F-68 and 2 ng/μL of sssDNA to serially dilute the DNase I treated AAV samples. (4) Employ a constant dPCR instrument to reduce interinstrument variability. (5) Thermal capsid lysis is performed by the predenaturation step (95 °C, 10 min) before the PCR amplification program to release genomic DNA. Do not perform thermal lysis prior to serial dilution.

**Figure 7:**
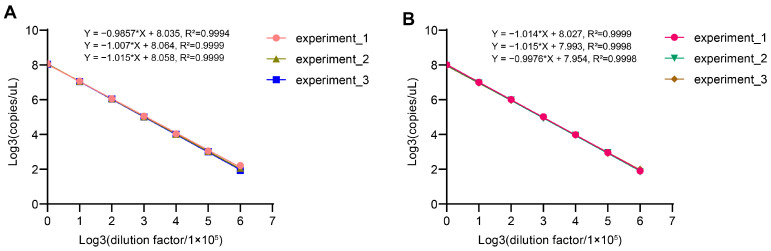
Validation of linearity of copy values against dilution factors in dPCR. The original copy values of genome DNA of AAV5 sample were reported by the dPCR instrument at different serial dilution factors in three experiments performed by technician 1 (**A**) and technician 2 (**B**), respectively. The fitted lines were created by plotting original copy values against the corresponding serial dilution factors at log3 scale.

To confirm the linearity, repeatability, and precision of the AAV genome titer determination protocol, each of the two technicians independently performed experiments on the AAV5 sample three times. As depicted in Figure 7, excellent linearity was presented by plotting copy values (copies/μL) determined from serial dilutions against the corresponding dilution factors at log3 scale; all R-square values were greater than 0.999. Additionally, there was substantial overlap among the different fitted regression lines of repeated experiments.

Moreover, we collected seven additional AAV-EGFP vectors and determined their genome titers with the optimized dPCR protocol for three independent tests to validate the applicability and precision of the dPCR protocol on multiple AAV serotypes. It was found that the RSD of titers from three tests was less than 5.00% for all these vectors (Table 2) regardless of how the samples were produced, indicating an acceptable variability in our protocol and tests, thereby demonstrating the reliability of the dPCR-based determination procedure for AAV genome titers.

## 3. Discussion

Ensuring the reliable and reproducible titration of rAAV preparations is crucial for preclinical studies and toxicological, clinical, and commercial dosing. The transition of genome titration methods from qPCR to dPCR has been driven by the improved accuracy and precision provided by this absolute quantification technology. However, variations in the operating procedures of dPCR titration can impact rAAV genome titers. In this study, we assessed the effects of different sample pretreatment conditions and dPCR instruments on rAAV genome titers. Additionally, we validated the optimized dPCR titration protocol across multiple AAV serotypes.

Digital PCR has been established for quantifying and characterizing rAAV genomes, with either extracted genome DNA or whole virions being partitioned into numerous independent parallel reaction compartments [6,7,8,9]. It is known that adeno-associated virions package either plus (sense)- or minus (antisense)-stranded genomes separately at a roughly 1:1 ratio [12]. Upon genome extraction, the plus-stranded and minus-stranded genomes can undergo strand annealing to form double-stranded species by Watson–Crick base pairing [12,13]; therefore, the accuracy of AAV genome titration, which is based on the genome extraction protocol, could be affected on account of introducing double-stranded DNA molecules and the subsequent determination of two viral genome copies as one copy. On the other hand, different viral DNA extraction protocols and commercial reagent kits, and even different batches of extraction experiments with the same reagents, can make it hard to ensure complete and consistent genome recovery. Our results confirm that the AAV genome titer measurement protocol involving genome extraction performed worse than that without extraction in terms of accuracy and precision. Thus, to avoid the influence of viral DNA extraction on genome titers, we adopted the procedure without genome extraction to evaluate the effects of several other potential risk factors on AAV genome titration.

Various dPCR instruments from different manufacturers have been used in the absolute quantification of nucleic acids. Although these instruments are generally based on microfluidics, they can differ in physical design and chemical reagents [14]. We observed close agreement among the results obtained using four instruments with the same batch of genome DNA extract, confirming the correct usage and reliable DNA quantification of each instrument. On that basis, different instruments reported significantly different titers for the same samples via the procedure without genome extraction, indicating that genome titers measured with different instruments could not be directly compared. Hence, we used one constant instrument to assess the effects of several potential impacting factors in the pretreatment of AAV samples, aiming to identify key operation details leading to variations and improving the reproducibility of AAV genome titer determination by dPCR.

AAV5 is the most stable serotype, with a melting temperature of 89.2 °C [11]. We tested the effect of DNase inactivation by heating the AAV5 sample to 80 °C after digesting. This temperature is distant from the melting curve of AAV5, making it a safe temperature to inactivate DNase only without disrupting viral capsids. The results show thermal inactivation of DNase I led to a lower genome titer in the AAV5 sample, suggesting that DNase I inactivation is unnecessary in the nonextraction pretreatment procedure. The DNase I will be diluted serially to a low concentration that is far from the working concentration. In agreement with previous studies [6,7], we also found and proved with a wider concentration range that different concentrations of Pluronic F-68 in dilution buffer do not result in AAV genome titer variations, but Pluronic F-68 remains an essential component [6]. In contrast, sheared salmon sperm DNA (sssDNA) is not indispensable for genome titer determination and does not affect the dilution buffer.

According to our results, AAV capsid lysis before serial dilution led to large variation with a greater than ten-fold decrease in genome titers; thus, the early capsid lysis prior to dilution was the primary risk factor causing the most substantial titer variation in this study. It is crucial to perform capsid lysis of viral vectors after serial dilution in dPCR titration, while capsid lysis carried out before assembling dPCR reactions or in reaction partitions in the dPCR instrument presented no obvious differences in titer determination [7].

In summary, titration without genome extraction, performing capsid lysis after dilution of AAV samples, and minimizing the freeze–thawing cycles of AAV samples can improve the accuracy and precision of AAV genome quantification by dPCR assay. DNase I treatment conditions, different concentrations of Pluronic F-68, and the addition of sssDNA or not in dilution buffer are not key factors leading variations in AAV genome titration. Additionally, different instrument platforms can give divergent quantification results for the same AAV sample, highlighting that variations coming from instruments should be noted. These findings are beneficial to reducing variability and improving the reproducibility of dPCR titration for the AAV genome.

## 4. Materials and Methods

### 4.1. RAAV Materials

AAV5-EGFP and AAV8-EGFP samples used in this study were retained samples in the division of recombinant drugs of the National Institutes for Food and Drug Control and were produced using a triple transfection platform. For the samples used in verification, the AAV2, AAV5 (a), AAV6, AAV8, and AAV9 (a) were produced by a triple plasmid transfection platform and purchased from VectorBuilder; the AAV5 (b) and AAV9 (b) were produced by baculovirus vectors in insect cell (rBV/Sf9) systems and purchased from GeneCradle.

### 4.2. Primers and Probes

PCR primers and probes were designed for the GOI (EGFP). Forward Primer: 5′- GCTGGAGTACAACTACAAC-3′; Reverse Primer: 5′-TGGCGGATCTTGAAGTTC-3′; Probe: 5′-VIC-CTTGATGCCGTTCTTCTGCTTGT-MGB-3′.

### 4.3. AAV Genome Extraction

AAV genomes were extracted with High Pure Viral Nucleic Acid Kit (Roche, Mannheim, Germany, cat# 11858874001). A total of 5 μL of undiluted AAV vectors was taken and added to 195 μL of TE buffer supplemented with 0.1% Pluronic F-68 (Gibco, New York, NY, USA, cat# 24040032) for a total volume of 200 μL as initial input of extraction with the kit; subsequent steps were carried out following the manufacturer’s instructions.

### 4.4. AAV Genome Titration by dPCR

The testing AAV sample was first treated with DNase I to digest unencapsidated DNA or residual plasmid DNA. The digestion reaction system included nuclease-free water (Invitrogen, Carlsbad, CA, USA, AM9937) 38 μL, 10× DNase I Reaction Buffer (NEB, Beverly, MA, USA, M0303L) 5 μL, DNase I (RNase-free) (NEB, M0303L) 2 μL, AAV sample 5 μL. Gently pipette and mix 10–15 times without introducing bubbles. Note not to vortex; otherwise, enzyme activity would be impacted. The reaction mix was incubated at 37 °C for 30 min, except for the test for the effect of DNase treatment time in this study. The DNase I treatment step served as the first ten-fold dilution of the AAV sample; subsequently, the DNase-treated AAV sample was serially diluted to 1 × 10^−5^ of the original concentration. Following this, a three-fold serial dilution based on this concentration was performed with the purpose of avoiding too wide a concentration range that deviates from the suitable range for dPCR detection. The dilution buffer we used was GeneAmp 10× PCR Buffer II and MgCl2 (25 mM) (Applied Biosystems, Carlsbad, CA, USA, cat# 4379878) with the addition of 0.1% Pluronic F-68 (100×, 10%) (Gibco, cat# 24040032) and 2 ng/μL sheared salmon sperm DNA (sssDNA) (Invitrogen, AM9680), except for the tests for buffer additive components in this study. Then, we prepared PCR mix with the diluted samples; the PCR reaction system for each loading well on a disposable digital PCR plate in the digital PCR kit (Applied Biosystems, A52688) contained Absolute Q Digital PCR Master Mix (5×) (Applied Biosystems, A52490) 2 μL, 10 μM Forward Primer 0.8 μL, 10 μM Reverse Primer 0.8 μL, 10 μM Probe 0.4 μL, nuclease-free water 1 μL, and 5 μL of each diluted AAV sample. The dPCR assay was conducted with QuantStudio Absolute Q instrument (Applied Biosystems), while other brands of instruments were used according to the manufacturer’s instructions when we evaluated the consistency and diversity of instruments. A heating stage at 95 °C for 10 min was set before the PCR amplification program to thermally lyse the AAV capsids and release genome DNA as templates in subsequent PCR: 50 cycles of 95 °C × 15 s, 55 °C × 1 min; 4 °C hold. DNA low-bind Eppendorf tubes and MAXYMum Recovery pipette tips were used throughout the operation procedure.

### 4.5. Statistical Analysis

Statistical analysis and graphical representation were performed using GraphPad Prism V9.5.1 software. A *p*-value of less than 0.05 was considered statistically significant. RSD % = 100% × standard deviation (SD)/average.

## Figures and Tables

**Table 1:** AAV genome titers determined from extracted genome DNA or samples without extraction.

	Serotype	Experiment_1	Experiment_2	Experiment_3	Avg (vg/mL)	RSD (%)
Extraction	AAV5	1.05 × 10^12^	1.02 × 10^12^	9.68 × 10^11^	1.01 × 10^12^	4.06
	AAV8	9.11 × 10^12^	1.31 × 10^13^	7.58 × 10^12^	9.92 × 10^12^	28.54
Without extraction	AAV5	1.40 × 10^12^	1.38 × 10^12^	1.37 × 10^12^	1.38 × 10^12^	1.24
	AAV8	1.00 × 10^13^	9.16 × 10^12^	9.28 × 10^12^	9.49 × 10^12^	4.99

One AAV5 sample and one AAV8 sample were tested three times using protocols with genome extraction and without extraction, respectively. Assay precision was determined by the RSD of 3 tests. “vg/mL” stands for viral genomes per milliliter.

**Table 2:** Validation of the precision of AAV genome titration across multiple serotypes.

Serotype	Test_1	Test _2	Test _3	Avg (vg/mL)	RSD (%)
AAV2	1.36 × 10^13^	1.37 × 10^13^	1.41 × 10^13^	1.38 × 10^13^	1.96
AAV5(a)	1.15 × 10^13^	1.08 × 10^13^	1.19 × 10^13^	1.14 × 10^13^	4.76
AAV6	8.47 × 10^12^	9.01 × 10^12^	8.30 × 10^12^	8.59 ×10^12^	4.32
AAV8	1.45 × 10^13^	1.48 × 10^13^	1.53 × 10^13^	1.49 × 10^13^	2.66
AAV9(a)	8.70 × 10^12^	9.07 × 10^12^	8.33 × 10^12^	8.70 × 10^12^	4.25
AAV5(b)	3.30 × 10^13^	3.36 × 10^13^	3.36 × 10^13^	3.34 × 10^13^	1.04
AAV9(b)	3.37 × 10^13^	3.32 × 10^13^	3.21 × 10^13^	3.30 × 10^13^	2.41

AAV samples of multiple serotypes were tested three times for genome titers using the optimized dPCR assay. The lower cases in the brackets after serotype names are used to distinguish samples; the two AAV5 samples, as well as the two AAV9 samples, were different samples, and they were produced with different packaging systems. The AAV5 and AAV8 samples here were different from those used before. Assay precision was determined by the RSD of 3 tests.

## Data Availability

Data are contained within the article and Supplementary Material.

## Supplementary Materials

The following supporting information can be downloaded at https://www.mdpi.com/article/10.3390/ijms25105149/s1.

## References

[1] Bulcha J.T., Wang Y., Ma H., Tai P.W.L., Gao G. (2021). Viral vector platforms within the gene therapy landscape. Signal Transduct. Target. Ther..

[2] Wang D., Tai P.W.L., Gao G. (2019). Adeno-associated virus vector as a platform for gene therapy delivery. Nat. Rev. Drug Discov..

[3] Wang Y., Menon N., Shen S., Feschenko M., Bergelson S. (2020). A qPCR Method for AAV Genome Titer with ddPCR-Level of Accuracy and Precision. Mol. Ther. Methods Clin. Dev..

[4] Dobnik D., Kogovsek P., Jakomin T., Kosir N., Tusek Znidaric M., Leskovec M., Kaminsky S.M., Mostrom J., Lee H., Ravnikar M. (2019). Accurate Quantification and Characterization of Adeno-Associated Viral Vectors. Front. Microbiol..

[5] Lock M., Alvira M.R., Chen S.J., Wilson J.M. (2014). Absolute determination of single-stranded and self-complementary adeno-associated viral vector genome titers by droplet digital PCR. Hum. Gene Ther. Methods.

[6] Sanmiguel J., Gao G., Vandenberghe L.H. (2019). Quantitative and Digital Droplet-Based AAV Genome Titration. Methods Mol. Biol..

[7] Prantner A., Maar D. (2023). Genome concentration, characterization, and integrity analysis of recombinant adeno-associated viral vectors using droplet digital PCR. PLoS ONE.

[8] Suoranta T., Laham-Karam N., Yla-Herttuala S. (2021). Optimized Protocol for Accurate Titration of Adeno-Associated Virus Vectors. Hum. Gene Ther..

[9] Zanker J., Lazaro-Petri S., Huser D., Heilbronn R., Savy A. (2022). Insight and Development of Advanced Recombinant Adeno-Associated Virus Analysis Tools Exploiting Single-Particle Quantification by Multidimensional Droplet Digital PCR. Hum. Gene Ther..

[10] Meierrieks F., Kour A., Patz M., Pflanz K., Wolff M.W., Pickl A. (2023). Unveiling the secrets of adeno-associated virus: Novel high-throughput approaches for the quantification of multiple serotypes. Mol. Ther. Methods Clin. Dev..

[11] Bennett A., Patel S., Mietzsch M., Jose A., Lins-Austin B., Yu J.C., Bothner B., McKenna R., Agbandje-McKenna M. (2017). Thermal Stability as a Determinant of AAV Serotype Identity. Mol. Ther. Methods Clin. Dev..

[12] Mayor H.D., Torikai K., Melnick J.L., Mandel M. (1969). Plus and minus single-stranded DNA separately encapsidated in adeno-associated satellite virions. Science.

[13] Nakai H., Storm T.A., Kay M.A. (2000). Recruitment of single-stranded recombinant adeno-associated virus vector genomes and intermolecular recombination are responsible for stable transduction of liver in vivo. J. Virol..

[14] Tan L.L., Loganathan N., Agarwalla S., Yang C., Yuan W., Zeng J., Wu R., Wang W., Duraiswamy S. (2023). Current commercial dPCR platforms: Technology and market review. Crit. Rev. Biotechnol..

