# Supplementary material for: Assessment of Key Factors Impacting Variability in AAV Vector Genome Titration by Digital PCR
Source: Int J Mol Sci. 2024 May 9;25(10):5149. doi: 10.3390/ijms25105149 (PMC11121146; doi:10.3390/ijms25105149)
Supplement: Supplementary file 1 [file ijms-25-05149-s001.zip › ijms-2970611-supplementary.pdf]

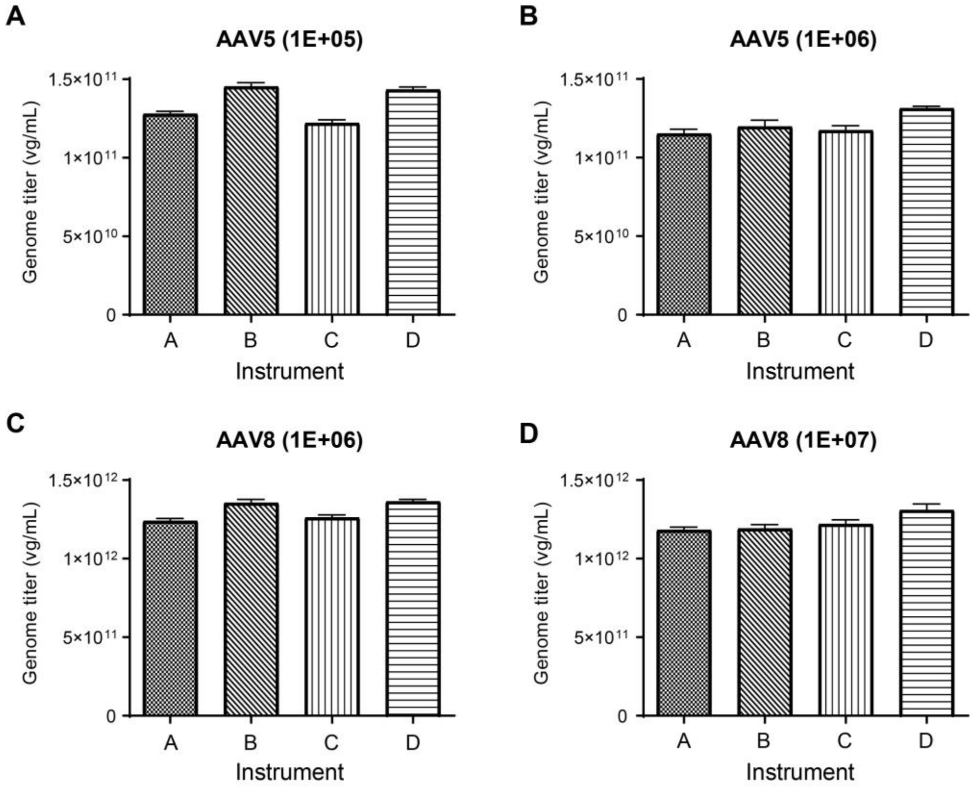

Figure S1. Consistency across dPCR instruments tested with extracted genome of AAV5 and AAV8

The genome titers of AAV5 (A,B) and AAV8 (C,D) samples determined by four brands of instruments with the same batch of genome extractive, both two samples were diluted and tested at two dilution factors. For each instrument, three experiments were performed for each AAV genome at each dilution factor, and three technical replicates were set, resulting in total nine data points per column. Data are presented as mean with standard deviation (SD).
